# Experimental and numerical evaluation of the dust suppression efficiency of an innovative vortex pneumatic fog screen dust control device

**DOI:** 10.1038/s41598-024-64140-2

**Published:** 2024-06-11

**Authors:** Shuaishuai Ren, Deji Jing, Shaocheng Ge, Yinuo Chen, Xiangxi Meng, Ping Chang

**Affiliations:** 1 College of Safety Science and Engineering, Liaoning Technical University, Fuxin, 123000 China; 2 Key Laboratory of Mine Thermodynamic Disasters and Control of Ministry of Education (Liaoning Technical University), Fuxin, 123000 China; 3 WA School of Mines: Minerals, Energy and Chemical Engineering, Curtin University, Kalgoorlie, WA 6430 Australia; 4 College of Safety and Emergency Management Engineering, Taiyuan University of Technology, Taiyuan, 03000 China

**Keywords:** Vortex pneumatic fog screen, Optimal working condition, Numerical simulation, Droplet size, Dedusting performance, Health occupations, Engineering, Risk factors

## Abstract

To effectively control the dust generated by coal mining operations, a new type of cyclonic pneumatic mist curtain dust control device was developed. Using CFD software, numerical simulations were conducted on the internal airflow velocity field, the exit velocity of the cyclonic pneumatic mist curtain, and the mist droplet particle field of the curtain. Experiments were carried out to measure the spray coverage, droplet size, and the dust control performance of the model device. The results indicate that when the water pump supply pressure is 8 MPa, the fan supply wind speed is 12 m/s, and the nozzle installation angle is 75 degrees, the cyclonic pneumatic mist curtain dust control device model operates under optimal conditions. The effective coverage of the cyclonic mist curtain is 380 × 3300 mm, fully suppressing dust generation on one side of the curtain. An optimal dust removal distance of about 90 cm was determined. After installing the cyclonic pneumatic mist curtain dust control device, the average dust reduction efficiency for respirable dust reached 91.07%, and the overall dust reduction efficiency achieved 93.34%.

## Introduction

Dust production in fully mechanized mining faces typically ranges from 20 to 38%^1–3^. Often, the total dust concentration and respirable dust concentration in heading faces significantly exceed relevant national standards, such as the Coal Mine Safety Regulations^4,5^. As the level of coal mine mechanization continues to advance, both the mines and working faces are evolving toward larger scale, higher intensity, and increased mechanical automation, consequently leading to a rise in dust concentration in fully mechanized mining faces and other working environments^6–10^. This escalation situation poses a significant risk to the safety of coal mine operations and the health of the workers^11–13^.

**Figure 1:**
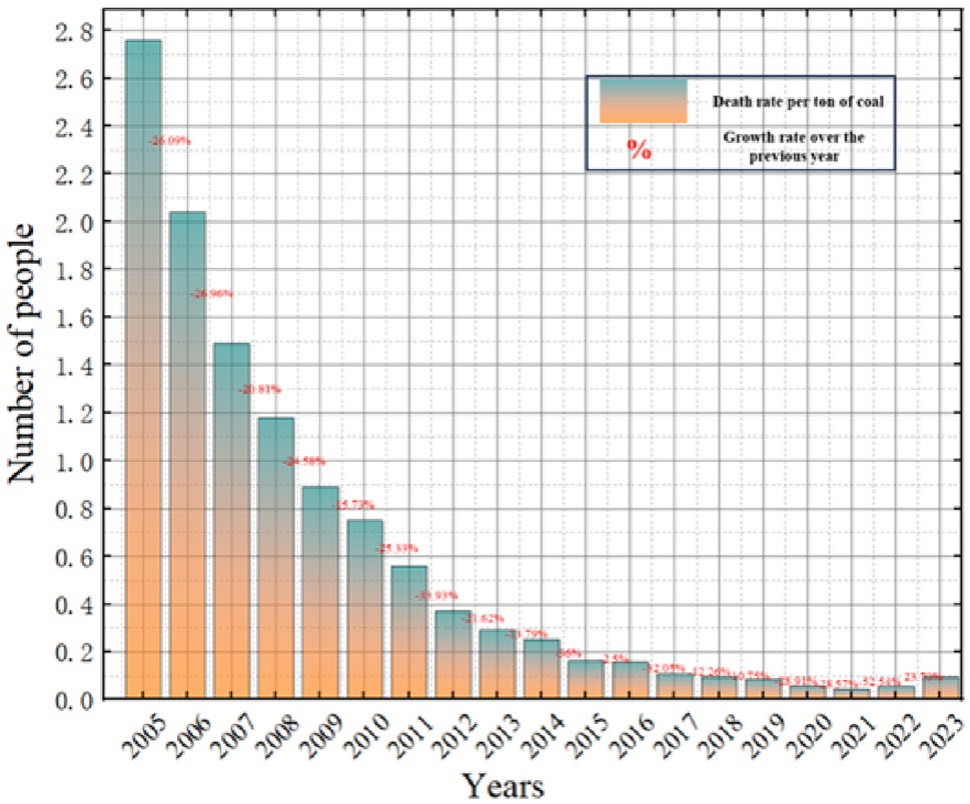
Mortality rate of million tons of coal in mine accidents from 2005 to 2023.

**Figure 2:**
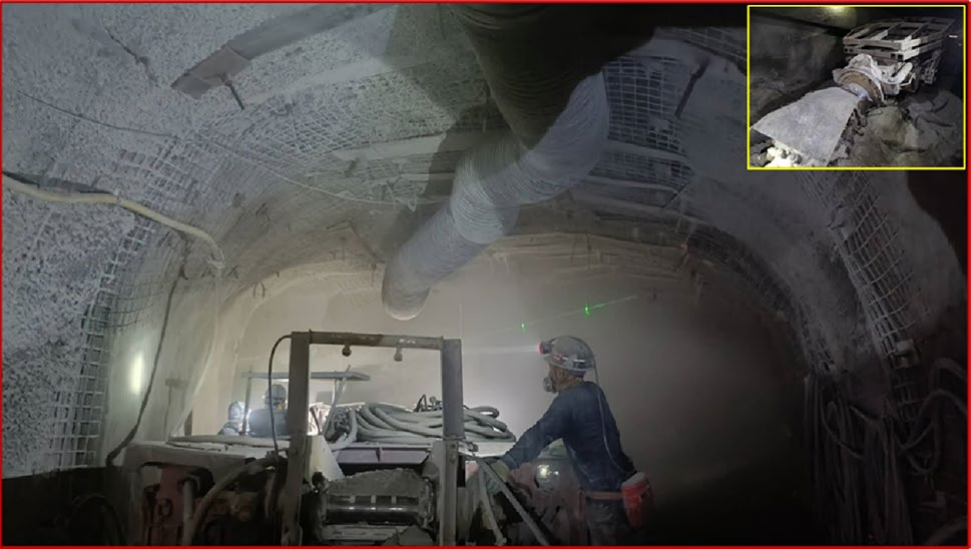
Dust generation during the excavation process.

In recent years, a significant decrease in the overall death toll from coal mine production accidents underscores the substantial and consistent improvements in mine safety^14,15^. By the year 2020, China reported 904,000 cases of occupational pneumoconiosis, with Coal Workers’ Pneumoconiosis (CWP) is accounting for more than 50% of these cases. Alarmingly, the fatality rate for CWP stands at approximately 2000 deaths annually, surpassing the number of fatalities from coal mine safety accidents^16–18^. The primary factor behind this high prevalence of mine pneumoconiosis cases is the inadequate attention given to occupational pneumoconiosis in coal and other enterprises, along with the ineffectiveness in controlling respirable dust emissions during coal mine production^19–21^. Figure 1 displays the death rate cases attributed to millions of tons of coal production-related accidents in China from 2005 to 2023, while Fig. 2 illustrates the level of dust pollution during the excavation process.

In the subterranean operations of a coal mine, a substantial amount of dust is generated during the excavation process, posing a significant pollution threat to the heading face^22,23^. The mitigation of dust pollution in underground mine heading faces has become a prominent and complex challenge that experts worldwide are closely monitoring. Currently, scholars from various countries have examined dust control technology for heading faces from various perspectives. Ye et al. employed the LES-VOF droplet collision probability algorithm, combined with other scholars' research on the generation and migration patterns of dust, to discuss the effects of spray pressure and effective spray distance on dust reduction. They found that spray pressures exceeding 5 MPa could achieve dust reduction efficiencies of around 90%^24,25^. Xu et al. used three-dimensional computational fluid dynamics simulations coupled with airflow to study the dust suppression effects of a cyclone separator dust control device, discovering that the optimal dust reduction occurred when the inlet wind speed was increased from 15 to 25 m/s^26^. Nie et al. researched the optimal air volume for dust curtain suppression technologies in controlling dust pollution dispersion, finding that the best suppression efficiency of 85% was achieved with an air volume of 90 m^3^/min^27^. However, these studies also noted challenges, such as the need for high pressures in high-pressure spraying to achieve effective dust control, substantial air pressures required for dust curtain devices, generally poor performance on fine particulates, and inflexible installation of the devices^28,29^. Currently, several studies have demonstrated that a combination of air curtain dust control and high-pressure spray dust control technology is a highly efficient means of effectively managing coal dust^30–36^. These studies encompass the optimization of working parameters for air curtain dust control devices to enhance their ease of installation and mobility, the secondary crushing of mist droplets, and improvements in capturing dust with small particle sizes^37,38^. Nonetheless, the existing dust control devices are unable to keep pace with the heading's tunnelling progress for continuous dust reduction operations^39,40^.

To address the aforementioned challenges, we have integrated wind curtain dust removal technology with high-pressure spray dust reduction technology. This device features a simple structure and operates with a low air volume, utilizing underground air ducts to supply air. It covers a large area, avoiding the direct use of fans, thereby enhancing the versatility of its installation. This approach creates a water fog curtain that fully envelops the dust source at the cutting head, effectively sealing and capturing respirable dust and further enhancing the underground working conditions in the coal mine. Through a combination of numerical simulations and experimental measurements, we've analyzed the formation mechanism of the vortex pneumatic fog screen and the process of combining wind and fog. As a result of these efforts, we have developed a vortex pneumatic fog screen dust control device that can operate simultaneously with the heading, improving dust control during excavation.

## Design of vortex pneumatic fog screen dust control device

**Figure 3:**
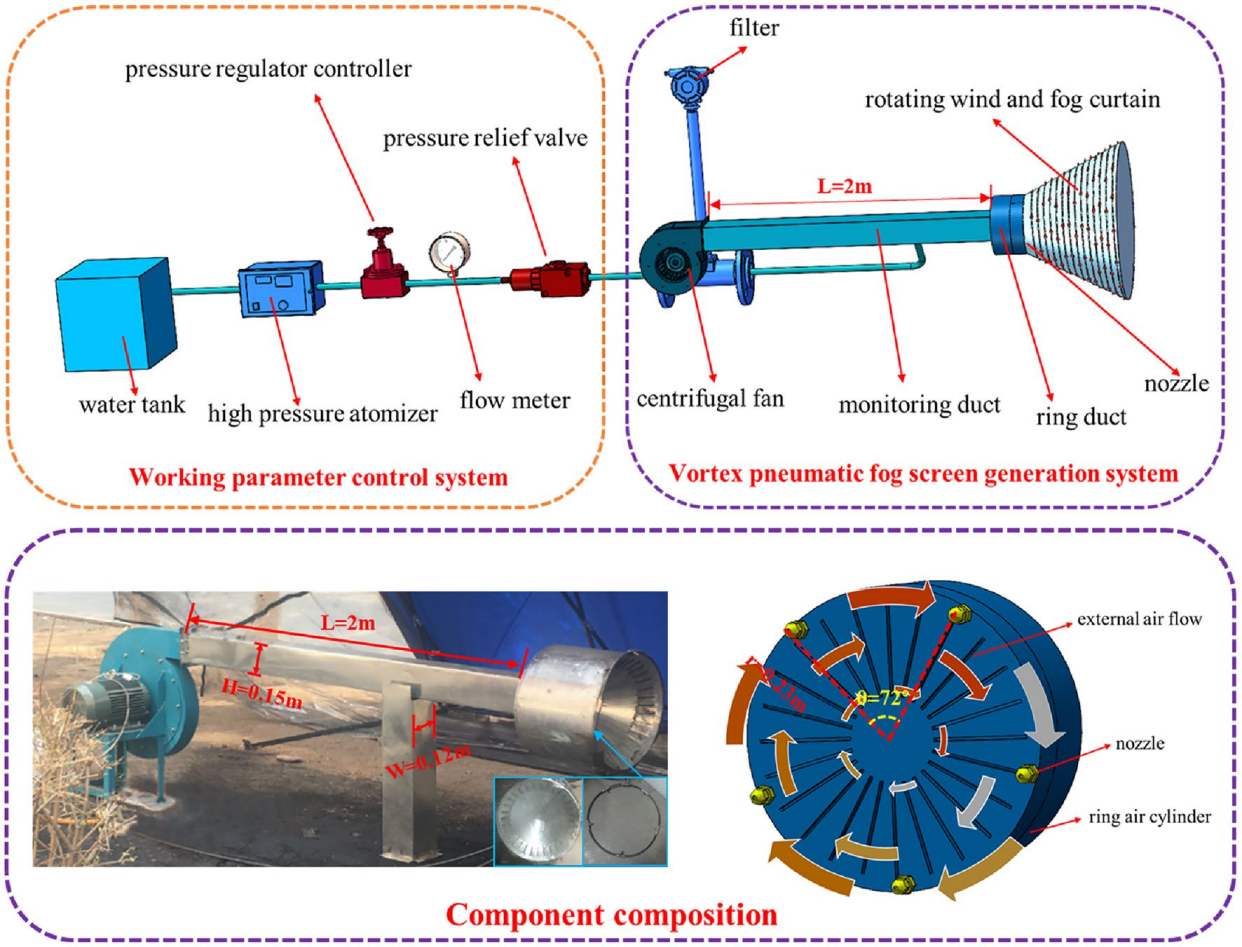
Location of pneumatic vortex spraying curtain.

To address the dust pollution in the aforementioned fully mechanized mining face, we propose the using a vortex pneumatic fog screen dust control device. As illustrated in Fig. 3, strategically positioned nozzles on the cutting head and evenly distributed along the air tube at specific angles work in unison. The droplets passing through the vortex air duct assist in creating an interconnected network of spiral fog curtains that effectively seal off the roadway and capture dust particles. Furthermore, the central region of the fog curtain generates a negative pressure zone directed towards the center of the inner annular air duct. This negative pressure compels the dusty airflow to interact once more with the fog curtain, leading to a secondary dust settling effect.

As depicted in Fig. 3, the primary components of the pneumatic vortex fog screen dust control device developed in this study include the annular air tube, high-pressure air flow ejector, and nozzles. The main equipment is introduced below:

1. Annular air duct:

The annular air duct functions as the transitional segment between the air inlet duct and the high-pressure air flow deflector. Its purpose is to partition and direct the airflow for the high-pressure air flow ejector.

(2) High-pressure airflow ejector:

The high-pressure air flow ejector consists of air outlets uniformly distributed on the inner surface of the annular air tube. These outlets guide the airflow inwards, crucial for generating the high-speed air jet. The high-pressure air flow ejector, uniformly positioned along the inner wall of the annular air tube, expels impacting airflow with tangential velocity components. This process ultimately forms a rotating air curtain wall with vortex impact momentum ahead of the air outlet.

(3) Spray nozzles:

The nozzles convert the pressure and water pressure energy within the annular pipe into kinetic energy. These annular nozzles serve as the pivotal elements for high-pressure spraying and serve as the primary points for atomization. The high-pressure nozzle model is CC-L, featuring an orifice diameter of 0.79 mm, a flow rate of 0.3 L/min, and an operating pressure of 8 MPa.

## Numerical simulation and experimental design *of vortex* pneumatic fog screen dust control device

### Mathematic model

The outlet velocity of the device, the airflow velocity field within the device, and the droplet particle field of the vortex pneumatic fog screen underwent numerical simulation using the control single variable method and CFD simulation software. The objective was to analyze the impact of various parameters on the atomization efficiency of the device, providing a theoretical foundation for vortex pneumatic fog screen dust control technology.

Turbulent flow is a common natural phenomenon. In most engineering scenarios, fluid flow is predominantly turbulent, with turbulence characteristics playing a significant role. In turbulent flow, the fluid exhibits disorder and chaos, even under constant boundary conditions, resulting in unpredictable flow characteristics such as varying velocities. As such, it becomes essential to simulate the airflow velocity distribution within the air duct generator^41–43^.

The Kelvin–Helmholtz (K–H) model is widely employed in the study of gas–liquid two-phase flow^44^. In this study, we utilized the incompressible k-ε turbulence model and a particle tracking model based on the K–H model to simulate the two-phase flow field in a vortex pneumatic fog curtain using CFD numerical simulation software. The governing equations of the K–H model describe the vortex spray and the secondary breakage of droplets within the vortex aerodynamic fog screen.

1 $$ \Lambda_{KH} = \frac{{9.02r_{0} \left( {1 + 0.45\sqrt Z } \right)\left( {1 + 0.4\left( T \right)^{0.7} } \right)}}{{\left( {1 + 0.865We_{g}^{1.67} } \right)^{0.6} }} $$

2 $$ \Omega_{KH} = \frac{{0.34 + 0.385We^{1.5} }}{{\left( {1 + Z} \right)\left( {1 + 1.4T^{0.6} } \right)}}\sqrt {\frac{\sigma }{{\rho_{l} r_{0}^{2} }}} $$

where Ω_*KH*_ is the maximum frequency, Λ_*KH*_ is the growth wavelength; the Weber number of the liquid is $We_{g} = \frac{{\rho_{g} U_{r}^{2} L}}{\sigma }$; U_*r*_ is the relative velocity difference between the liquid phase and gas phase; L is the feature length; σ is the surface tension coefficient of the liquid; ρg and ρf are the densities of the gas phase and liquid phase, respectively; Z is the Unsaiğ number, $Z = \frac{{\sqrt {We_{l} } }}{{Re_{l} }}$; The liquid Reynolds number is $Re_{l} = \frac{{\rho_{f} U_{r}^{2} r}}{\mu }_{f}$; $\mu_{f}$ is the viscosity of the liquid; and $T = Z\sqrt {We_{g} }$ is the Taylor number.

During the crushing process, the atomization rate of particles is calculated as follows:

3 $$ \frac{dr}{{dt}} = - \frac{{r_{0} - r_{KH} }}{{\tau_{KH} }},r_{KH} \le r_{0} $$

When r_KH_ is the radius of Kmuri H broken particles, the shear force is $\tau_{{{\text{KH}}}} = \frac{{3.788{\text{B}}_{{{\text{KH}}}} }}{{{\Lambda }_{{{\text{KH}}}} {\Omega }_{{{\text{KH}}}} }}$. B_KH_ is the empirical constant < 10, indicating that the atomization time of particles. The smaller the value, the earlier the decomposition time, and the faster the atomization speed. Then, the radius of the subdroplet produced when the mother droplet with a radius equal to rp breaks is

4 $$ {\text{r}} = \left\{ {\begin{array}{*{20}l} {B_{0} {\Lambda }_{{{\text{KH}}}} \le a} \hfill \\ {min\left\{ {\left( {\frac{{3\pi a^{2} U_{r} }}{{2{\Omega }_{KH} }}} \right)^{0.33} ,\left( {\frac{{3a^{2} {\Lambda }_{{{\text{KH}}}} }}{4}} \right)} \right\},B_{0} {\Lambda }_{{{\text{KH}}}} > a} \hfill \\ \end{array} } \right. $$

In the formula, B_0_ is a constant, taking 0.61, and Ur is the velocity difference between the gas and liquid phases.

### Physical model

The numerical simulation of the wind flow field employs the standard k-epsilon turbulence model, renowned for its high accuracy in calculating reverse pressure gradient flow fields. The Reynolds number associated with spiral spray dust control technology is substantial, with the inertia force significantly outweighing the fluid's viscous force.

The physical model of the three-dimensional scroll spray generator has been created using CFD simulation software. In real working conditions, it's observed that when the number of nozzles is less than 5, the vortex spray generator cannot generate a complete fog screen. Conversely, when the number of nozzles exceeds 5, the sprays produced by the vortex spray generator start interfering with one another, making it challenging to establish an effective fog curtain. Therefore, the high-pressure spray ring is comprised of five high-pressure nozzles, each positioned 72° apart. The airflow channel measures 2000 mm in length, 120 mm in width, and 150 mm in height, with the vortex spray head having a radius of 0.23 m.

**Figure 4:**
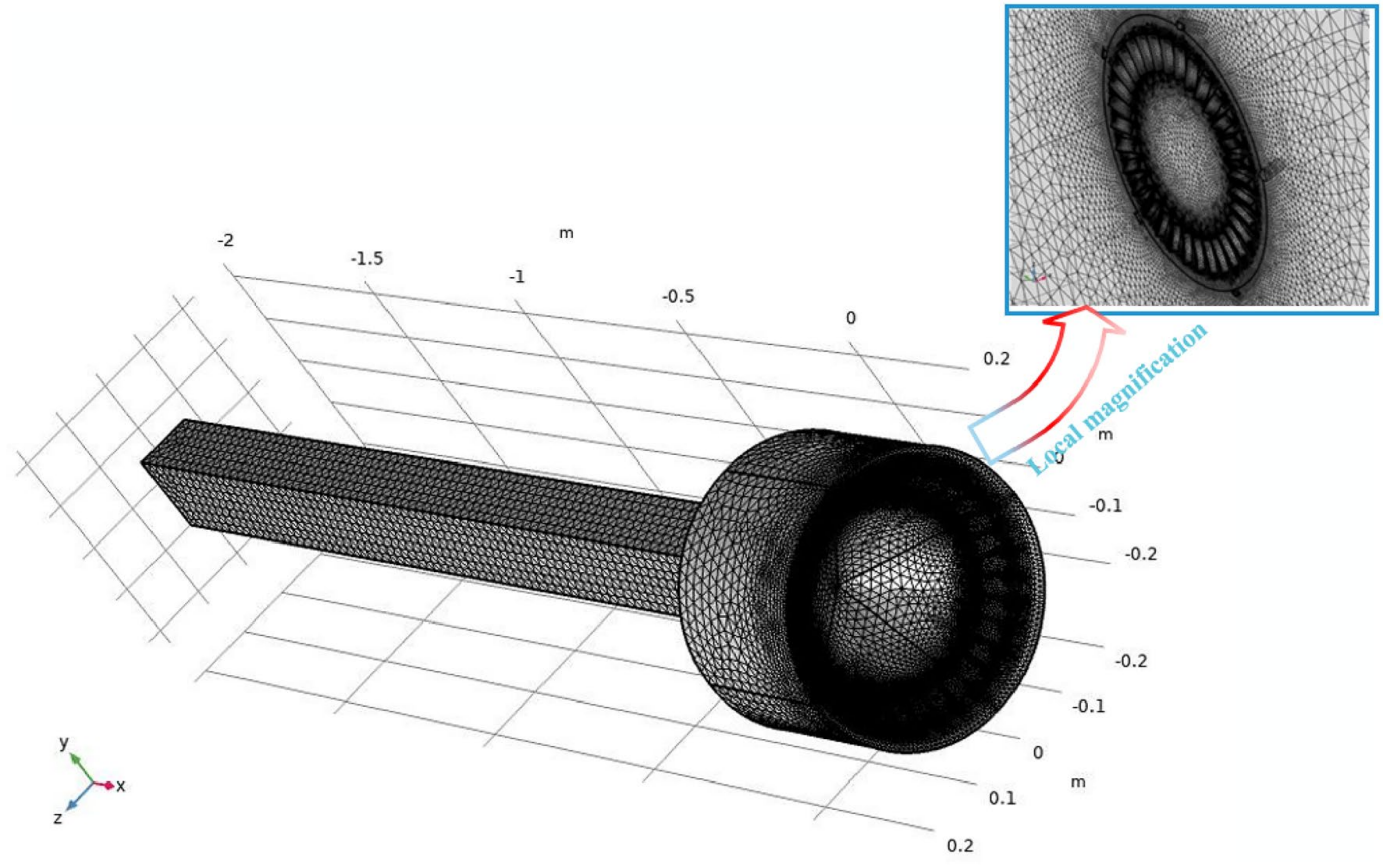
Grid drawing of vortex generator.

Meshing is a crucial aspect of numerical simulation as it directly impacts the accuracy and precision of the results, potentially leading to errors in numerical calculations. Figure 4 illustrates a physical model grid simulation diagram of the vortex pneumatic fog screen dust control device.

**Figure 5:**
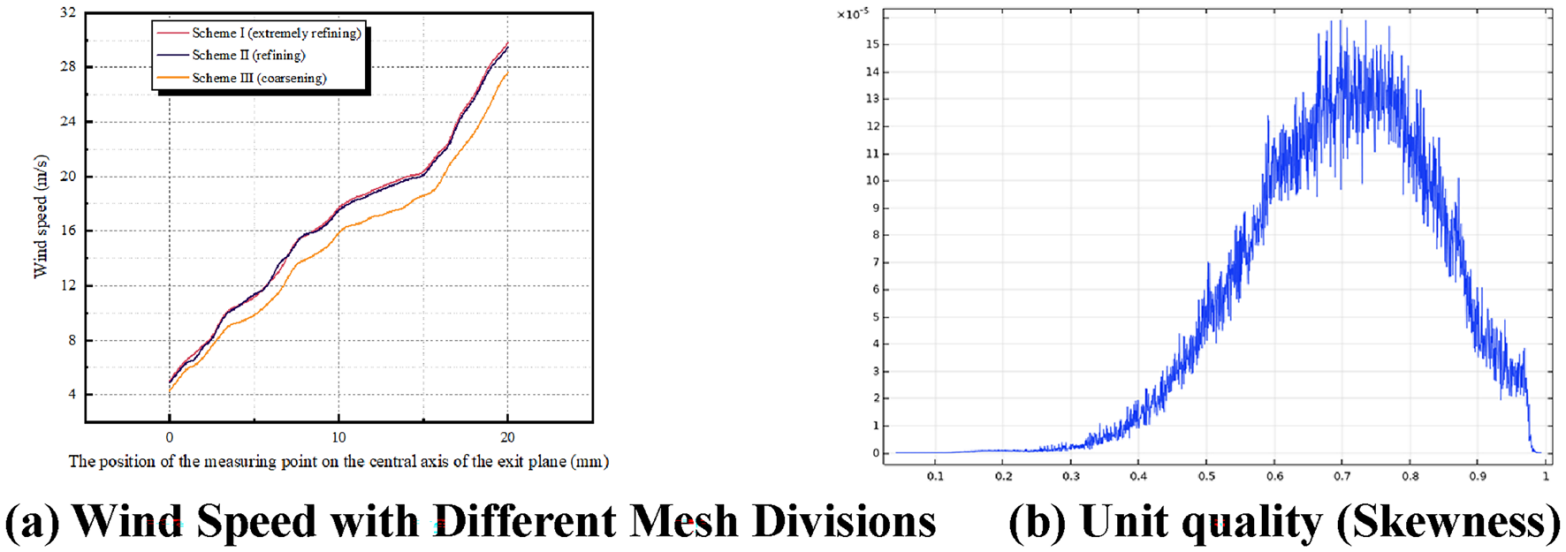
Mesh Division Results3.3 Model condition setting.

Grid independence is verified by comparing the computational results at different mesh densities. If the results stabilize with mesh refinement, the simulation can be considered grid-independent. Figure 5a shows the variation in the simulated wind speed at the head of the vortex airflow for three different meshing schemes. It is evident that Scheme II produces results that are not significantly different from those obtained with an ultra-fine mesh, thus Scheme II has been selected for the numerical simulation’s mesh division. This simulation utilized a total of 2,456,938 mesh elements, with an average element size of 0.6745 and a minimum size of 0.0374. The element quality distribution, depicted in Fig. 5b, falls within the range of 0.5 to 1, confirming the reliability of the mesh quality. Additionally, the correctness and reliability of the mesh division can be further validated through experimental verification.

According to the actual working conditions of the vortex pneumatic fog screen dust control device and the field data measurement, the relevant boundary conditions are set by using COMSOL software, as shown in Table 1.

**Table 1:** Boundary conditions and parameter values.

Parameter name	Parameter setting
Inlet wind speed (m s^−1^)	12
Outlet pressure (Pa)	0
Spray pressure (MPa)	8
Air density (kg m^−3^)	1.25
Dynamic viscosity of continuous phase (Pa s)	1.8 × 10^–5^
Diffusion coefficient of gas molecule (m^2 ^s^−1^)	2 × 10^–5^
Particle self-density (g cm^−3^)	1
Number of entry particles (a)	1000

### Experiment on coverage *of vortex* pneumatic fog screen

**Figure 6:**
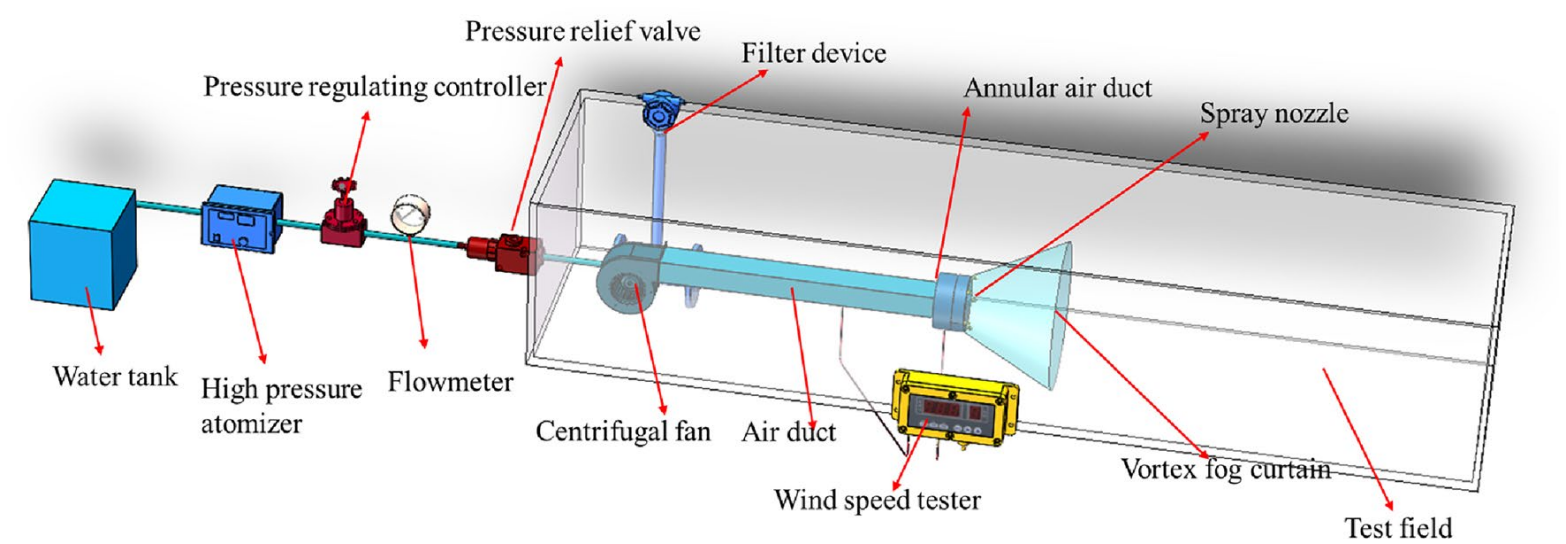
Schematic of experimental platform.

We measured the coverage area of the fog screen produced by the vortex pneumatic fog screen device. The experimental setup included a vortex fog curtain generating device consisting of a high-pressure spraying device, a centrifugal fan, a metal air supply pipe, and a vortex air flow generator. During the experiment, a thermal anemometer was used to measure the vortex wind speed. In this experiment, we employed the controlled variable method. To enhance the device's capability to capture small particle-size dust, we increased the water supply pressure when feasible, opting for a novel high-pressure spray system with a pressure setting of 8 MPa. We regulated the fan's blowing speed using a frequency converter and conducted tests to assess the spray coverage at various fan angles (ranging from 30° to 105°) at different wind speeds (0 m/s, 40 m/s, 60 m/s, 80 m/s, 100 m/s, and 120 m/s). Figure 6 shows a schematic diagram of the experimental platform.

### Experiment on measuring droplet size of vortex pneumatic fog screen

**Figure 7:**
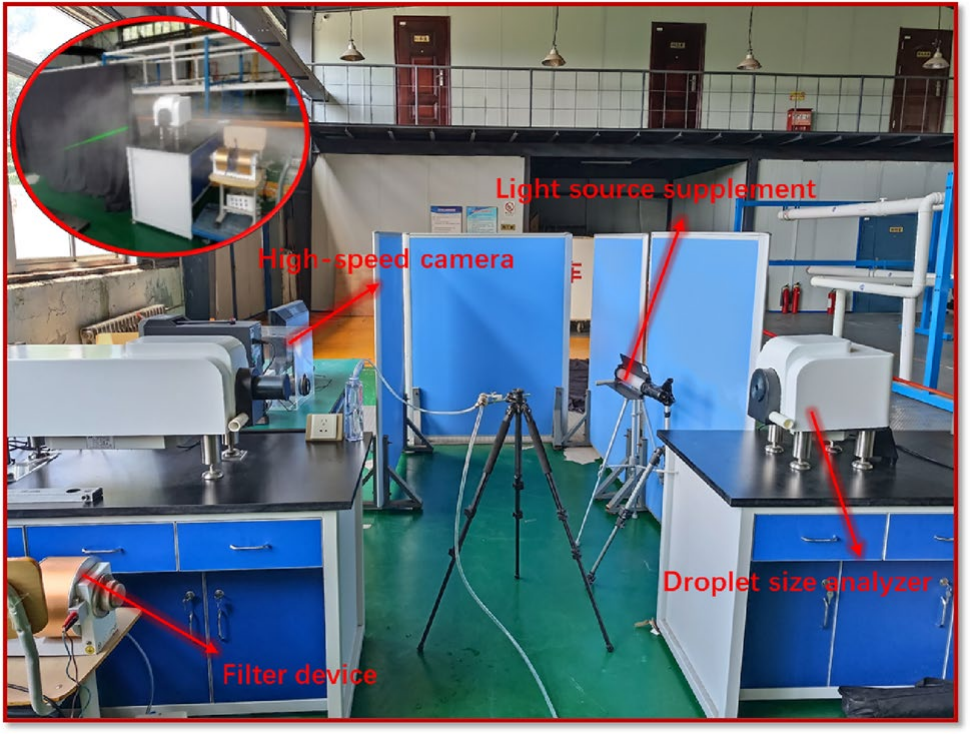
Droplet size testing platform.

Building upon the experiment regarding the coverage area of the vortex pneumatic fog screen, we maintained the pressure of the high-pressure spraying device at 8 MPa. We conducted tests to measure the spray diameter of the corresponding fan at a 75° angle and compared it with the performance of a traditional high-pressure spray (with the fan's wind speed set to 0 m/s). The experiment employed a Fraunhofer diffraction principle particle size analyzer for testing, and the droplet size testing platform is illustrated in Fig. 7.

The detailed experimental protocol is as follows:

1. Instrument calibration: The emitted laser beam must form a uniform, regular green spot at the receiver end. The beam should pass through the center of the Fourier mirror and the collimating lens and be perpendicular to the collimating lens.
2. Turn on the laser particle size analyzer and allow it to warm up for 10 to 15 min. Start the computer and launch the particle size analysis software.
3. Select "Background Measurement" from the "Test" menu, accumulate ten background measurements, then proceed to the energy spectrum test.
4. Adjust the fan speed and vary the spray distance to conduct control experiments. By adjusting the distance between the cyclonic pneumatic generator and the particle size analyzer, record the experimental data on changes in droplet size of the cyclonic pneumatic mist curtain at different distances. Conduct ten sets of experiments for each group and calculate the average data to ensure the accuracy of the final results.

### Experiment on dust reduction performance of a vortex pneumatic fog screen device

**Figure 8:**
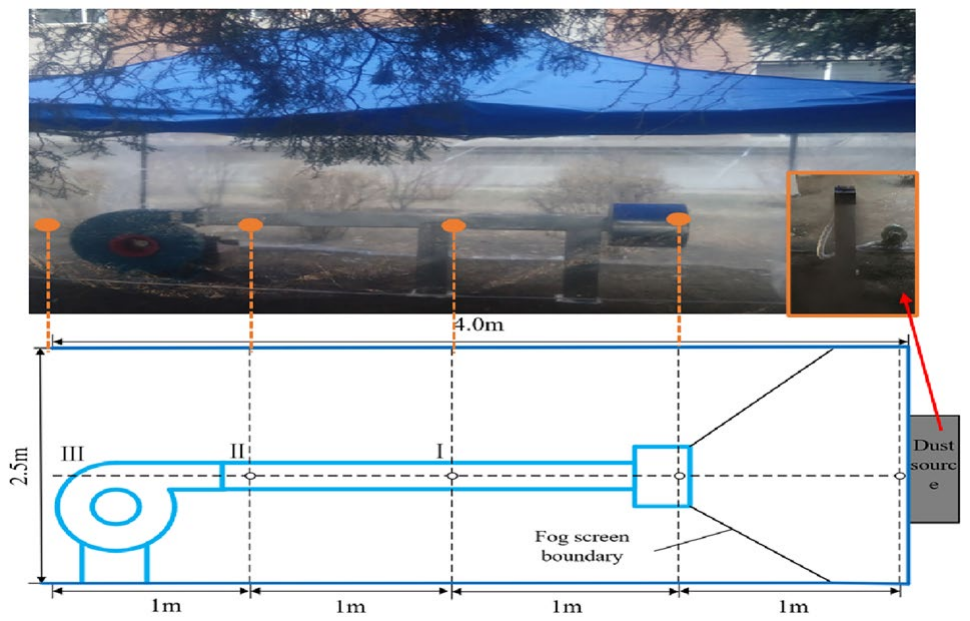
Survey point layout.

In order to investigate the dust reduction effectiveness of the vortex pneumatic fog screen dust control device within an underground driving roadway, we constructed an experimental shed on the ground. This shed has dimensions of 4 m in length, 3 m in width, and 2.5 m in height, serving as a simulation roadway. At the far end of this simulation roadway, dust emissions were simulated and contained, while the opening at the near end was utilized for testing the ventilation and dust control performance. The coal samples used in the experiment were bituminous coal from Wangjialing Coal Mine, owned by China Coal Huajin Group. To commence the experiment, coal blocks were crushed and ground into fine particles ranging in size from 60 to 2500 mesh (5.9 μm to 247 μm). This pulverized coal was then fed into a custom-made dust generator, which continuously produced dust at a rate of 10 g per second. Once the entire space inside the experimental shed was filled with dust, we activated the vortex pneumatic fog screen dust control device. The nozzle was set at a 75° installation angle, the fan operated at a wind speed of 12 m per second, and the water supply pressure was maintained at 8 MPa. Within the transverse section, three dust detection points were established, as illustrated in Fig. 8, which displays the layout of these survey points.

The dust concentration of the measuring point before and after opening the vortex pneumatic fog screen dust control device is measured, and the dust reduction efficiency of the vortex pneumatic fog screen dust control device is calculated according to Formula 5:

5 $$ \eta = \frac{{c_{1} - c_{0} }}{{c_{0} }} \times 100\% $$

In the formula, the average dust reduction efficiency is $\eta$—dust prevention measures, %. *c*_*0*_—average dust concentration after opening the vortex pneumatic fog screen, mg/m^3^. *c*_*1*_—-average dust concentration before opening the vortex fog screen, mg/m^3^.

## Results and discussion

### Simulation of airflow velocity field in vortex generator

**Figure 9:**
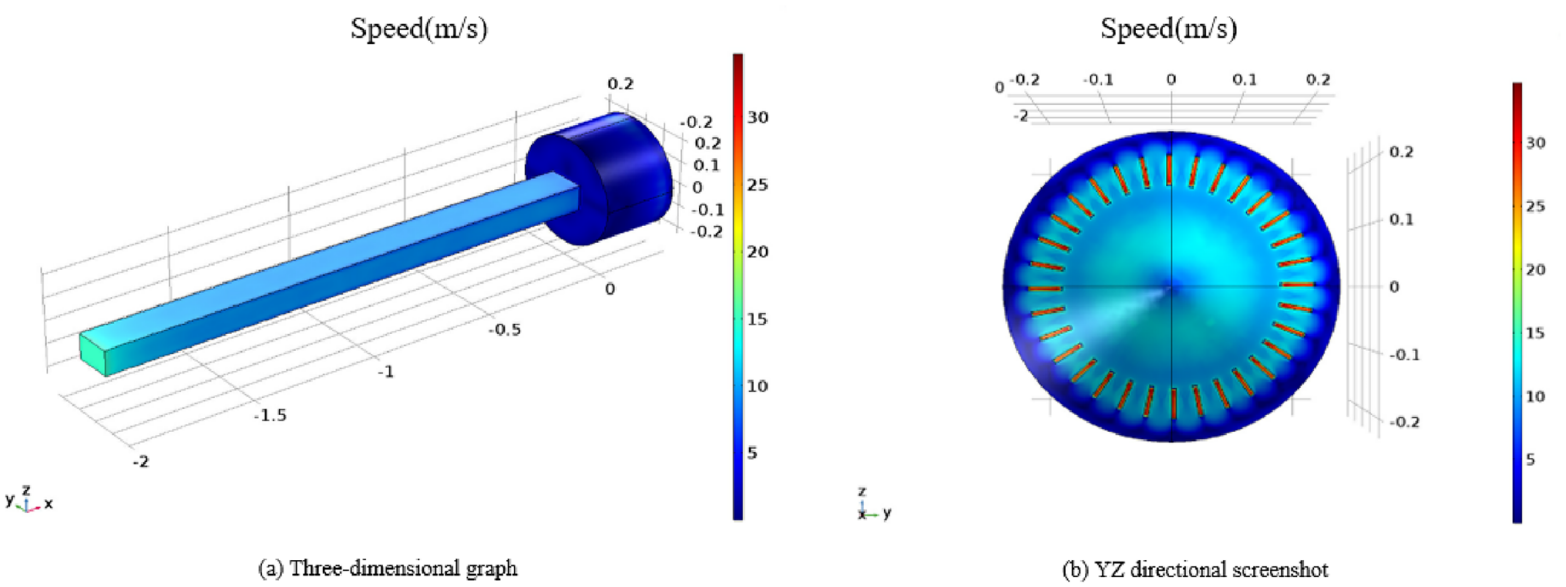
Velocity distribution of air duct.

**Figure 10:**
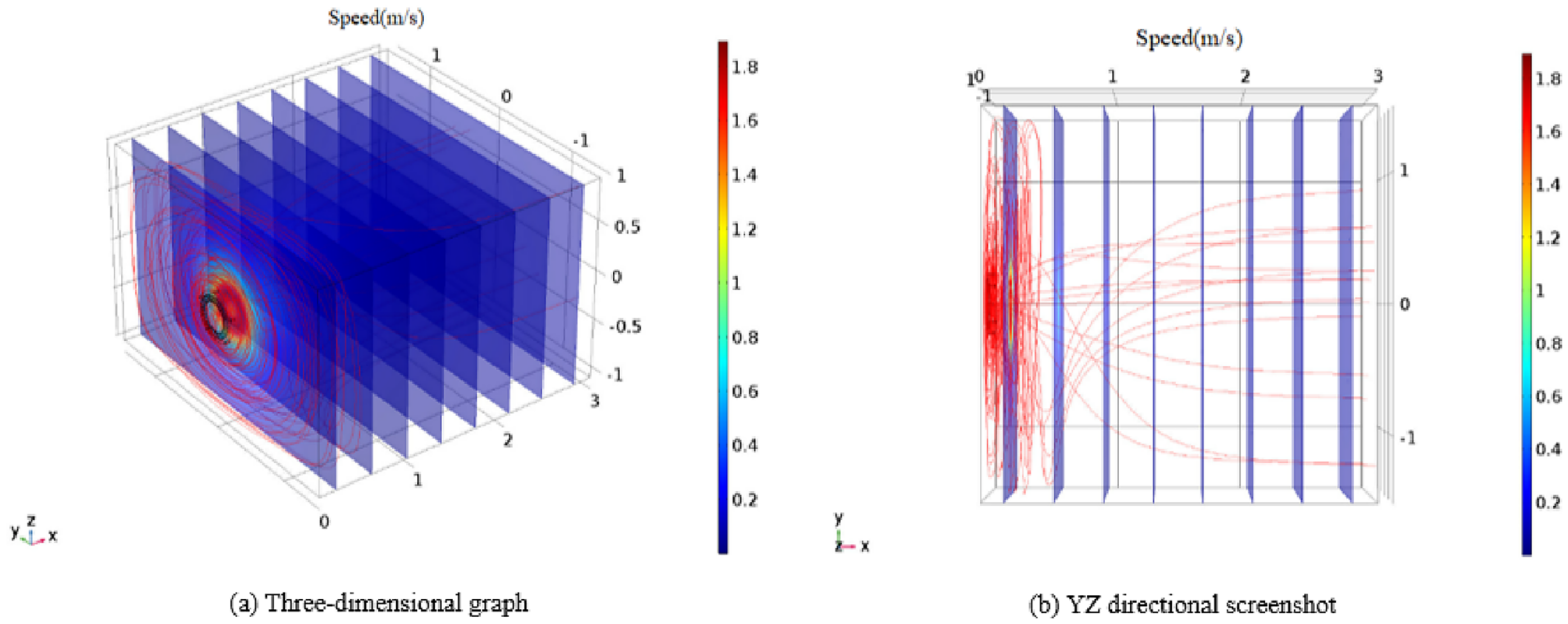
External air flow field of the device.

We conducted a simulation to analyze the airflow velocity field within the vortex generator. In order to prevent interference from the roadway airflow with the formation of the vortex pneumatic fog curtain during the actual operation of the device, it was necessary to set a higher wind speed for the pneumatic fog curtain. Based on the actual working conditions and the stable long-term operation capability of the centrifugal fan, we set the inlet wind speed to 12 m/s. Figure 9 illustrates the velocity distribution of the simulated airflow in different sections of the vortex generator, with the gradient zone on the right side of the numerical simulation chart representing the velocity. As depicted in Fig. 9, the inlet speed of 12 m/s results in an outlet wind speed of 30 m/s. Figure 10 presents the external wind flow field of the vortex generator.

As observed in the visual representation, the vortex airflow projects forward in a vortex-shaped manner, and its range progressively expands, gradually shaping a rotating wind curtain. This closely mirrors what was observed during the experimental phase. Figure 10 reveals that the airflow diminishes rapidly once it escapes through the 32 slits of the louvers. Within a distance of 0.3 m, the airflow velocity decreases to 1.2 m per second, and at 0.8 m, a minimal amount of airflow is directed along the x-axis.

### Exit velocity simulation of vortex pneumatic fog screen

We conducted a simulation to analyze the outlet velocity of the vortex pneumatic fog screen. In this simulation, the section boundary type for the airflow entering the calculation area was set as a velocity inlet, while the outlet was designated as a pressure outlet. The nozzle was positioned along the positive x-axis direction, and the particle type was specified as liquid drops, with water as the material and a spray pressure of 8 MPa.

**Figure 11:**
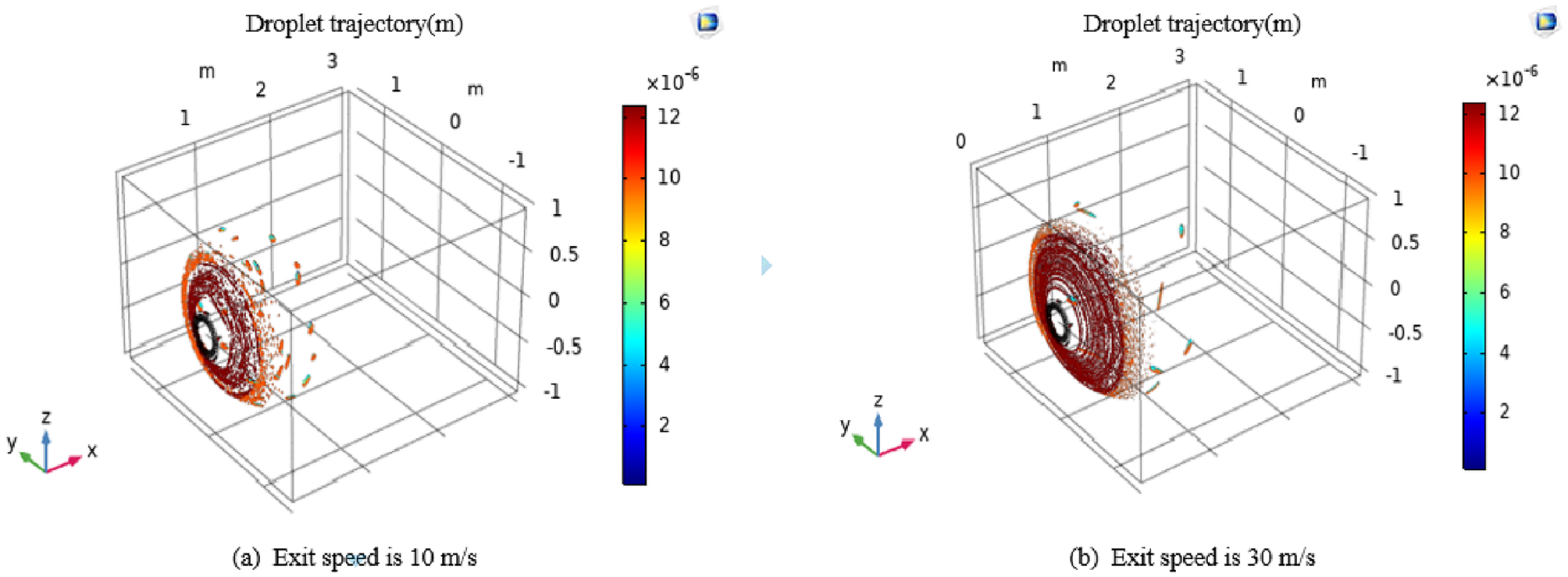
Droplet trajectory diagram at exit velocities of 10 m/s and 30 m/s.

As depicted in Fig. 11, the device's outlet velocity progressively transforms into a vortex-like fog screen once it surpasses 10 m/s. Furthermore, it is evident that the thickness of the vortex spray ring diminishes as wind speed increases. However, the cross-sectional area expands, resulting in greater spray coverage. It's important to highlight that for enhanced dust capture efficiency, it is advantageous to have a higher spray speed, allowing fog droplets to collide more vigorously with dust particles. Based on practical considerations, it was observed that when the airflow velocity of the vortex spray head reaches 35 m/s, it generates increased vibrations in the airflow channel. To ensure the device's long-term operational stability, an outlet velocity of 30 m/s has been defined as the optimal wind speed for this device. Two exit velocities were examined: 10 m/s and 30 m/s.

### Simulation of droplet particle field in vortex pneumatic fog screen

**Figure 12:**
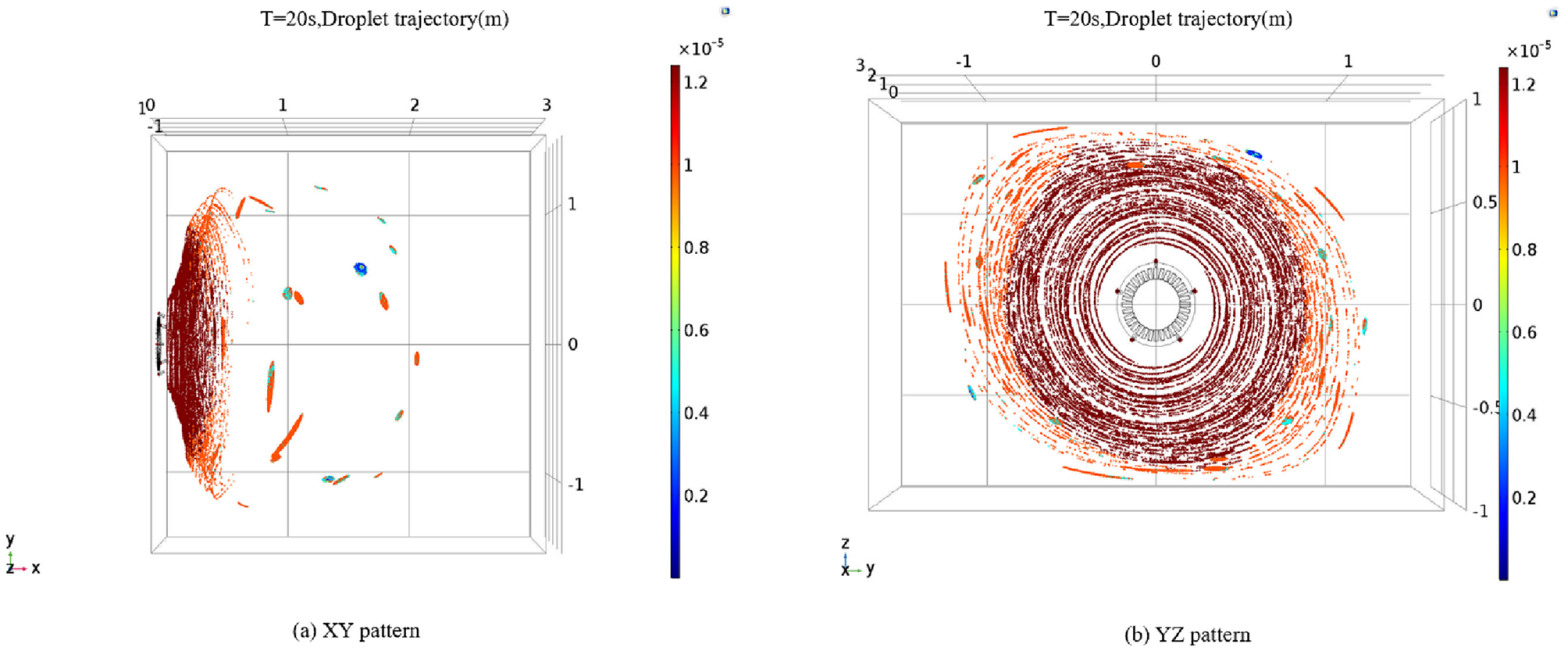
Trajectory diagram of vortex spray particles at nozzle angle of 60°

**Figure 13:**
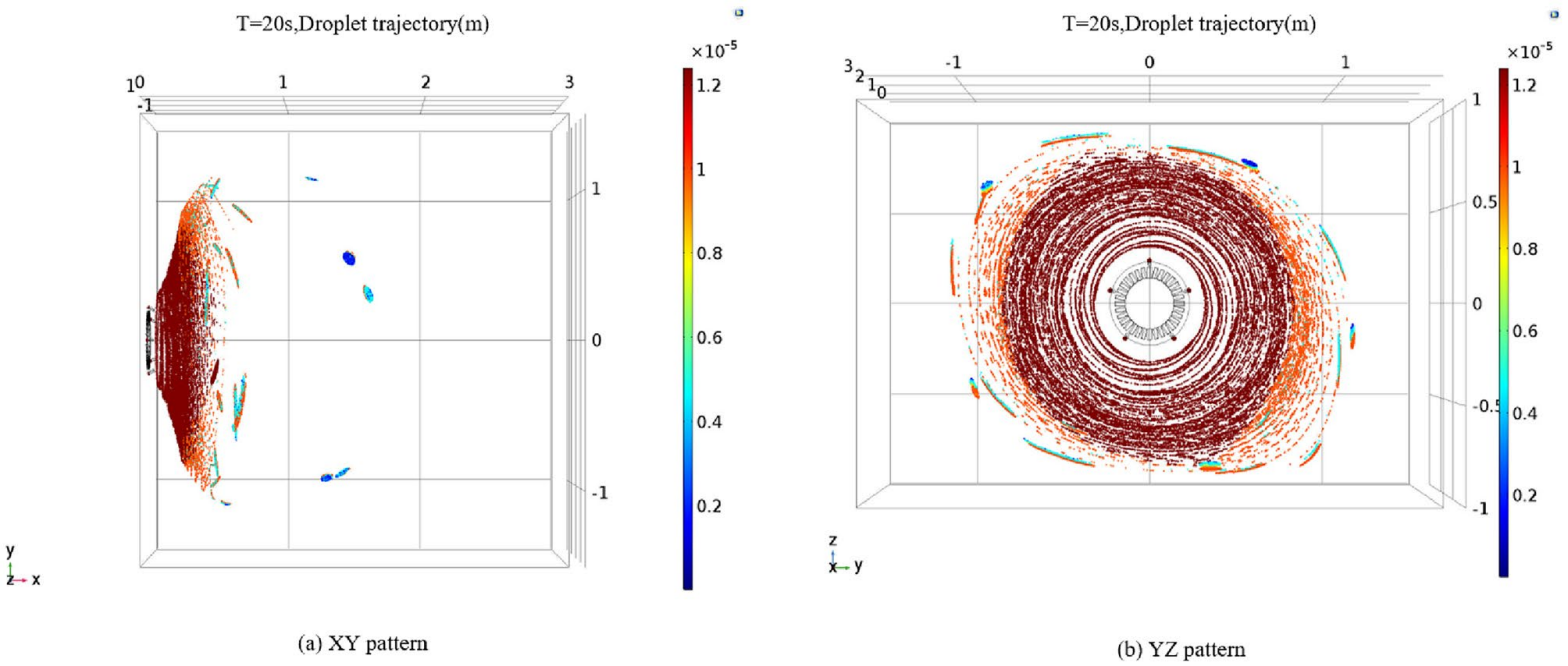
Trajectory diagram of vortex spray particles at nozzle angle of 75°

**Figure 14:**
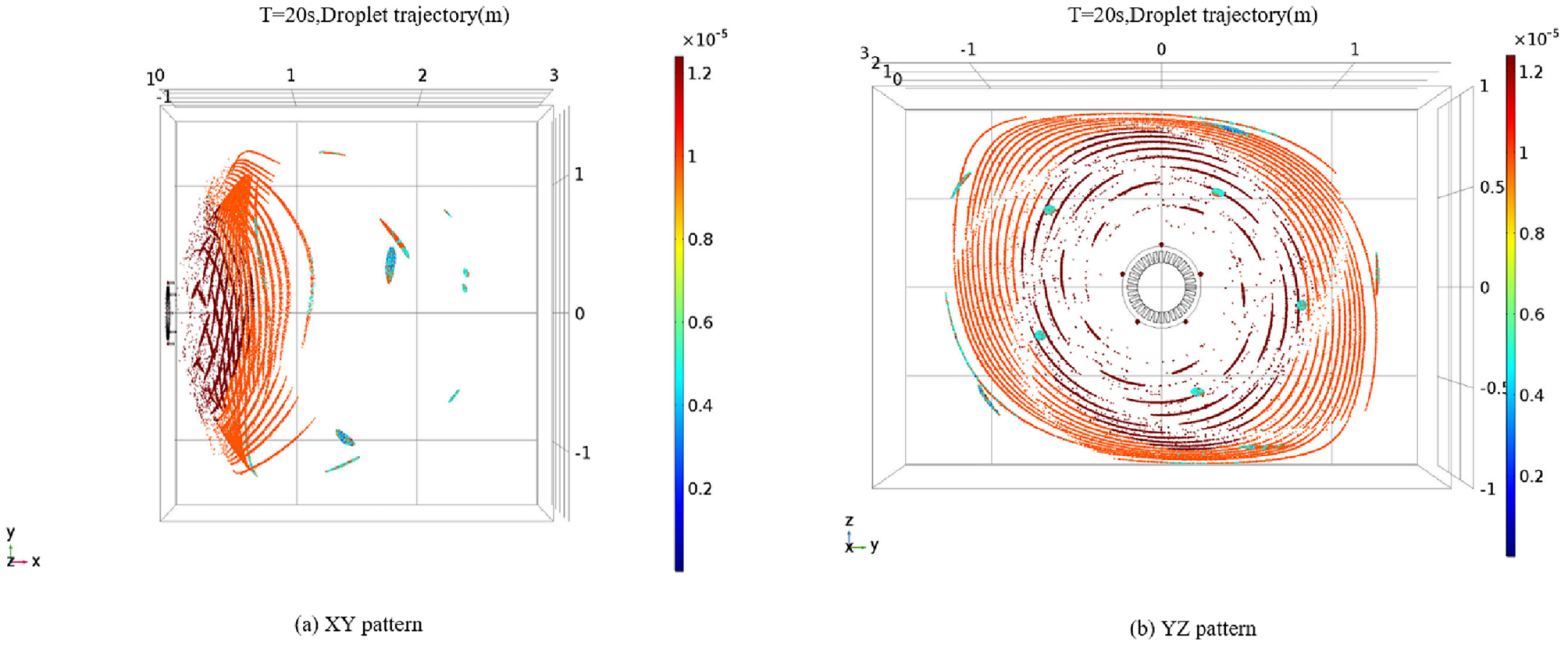
Trajectory diagram of vortex spray particles at nozzle angle of 90°

Following the adjustment of nozzle angles, we conducted simulations to observe the droplet particle spray process and obtain information regarding the range of droplet sizes and fog screen coverage. For these simulations, we maintained a wind speed of 30 m/s and an initial spray velocity of 30 m/s. Three nozzle angles, specifically 60°, 75°, and 90°, were chosen for the simulation. The results of the droplet particle field simulations for these three nozzle angles are presented in Figs. 12, 13 and 14. The numerical simulation charts include a gradient band on the right side to indicate the droplet sizes.

As illustrated in Figs. 12, 13 and 14, when the nozzles are installed at a 60° angle, the mist curtain moves forward in a circular shape with a diameter of 2.6 m, and the effective spray distance is 0.4 m, 20 s post-initiation. The droplet sizes range from 3 to 12 μm. Notably, the atomization effect of droplets produced by the vortex generator surpasses that of traditional high-pressure spraying. As the spray distance increases, the droplet size gradually decreases, with most droplets disappearing beyond a 0.4-m spray distance. Conversely, when the nozzles are set to a 75° angle, the effective spray distance remains at 0.4 m, with the mist curtain advancing in a circular shape with a diameter of 2.6 m, and droplet sizes roughly between 3 and 12 μm. At a spray distance of 0.2 m, the droplet diameter is approximately 12 microns. Beyond a spray distance of 0.4 m, the primary droplet diameter stabilizes at 6 μm, with most droplets disappearing after exceeding this threshold. This trend indicates that droplet size diminishes with increasing spray distance. It is noteworthy that when the nozzle angle is set to 90°, both the coverage area and spray distance slightly decrease compared to the 75° nozzle angle setting, with the mist curtain moving forward in a circular shape with a diameter of 2.5 m and an effective spray distance of 0.35 m. Additionally, high-speed airflow not only aids in the secondary breakup of droplets but also assists in capturing and controlling fugitive dust particles through generated negative pressure. This mechanism enhances dust suppression efficiency as smaller water droplets can more effectively combine with airborne dust particles. Since the high-speed airflow does not directly blow onto the dust source, its primary function is to facilitate droplet breakup, thereby preventing secondary dust generation. While ensuring effective dust reduction, it is essential to moderately reduce the concentration of mist to prevent an overly humid work environment. Excessively dense mist can also decrease visibility in the workplace, impacting safety and operational efficiency.

### Experiment on coverage of vortex pneumatic fog screen

**Figure 15:**
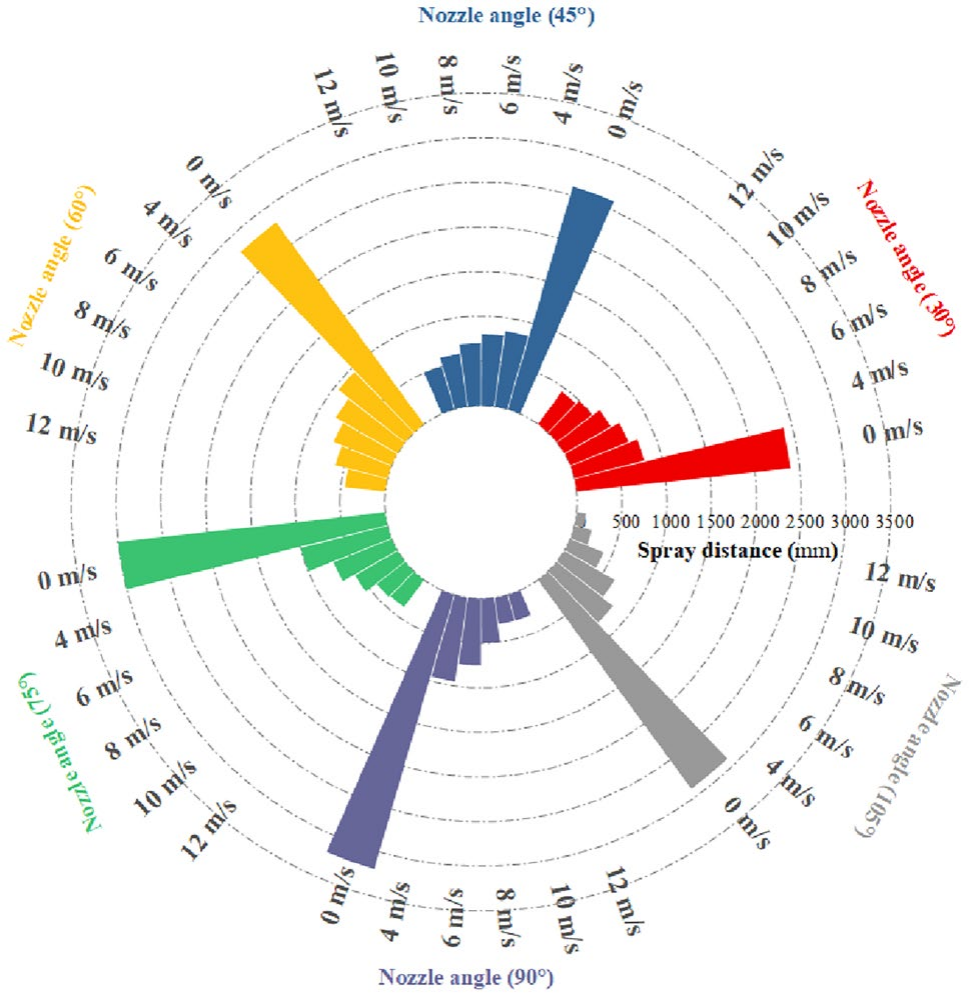
Variation law of effective spray distance of fog screen.

**Figure 16:**
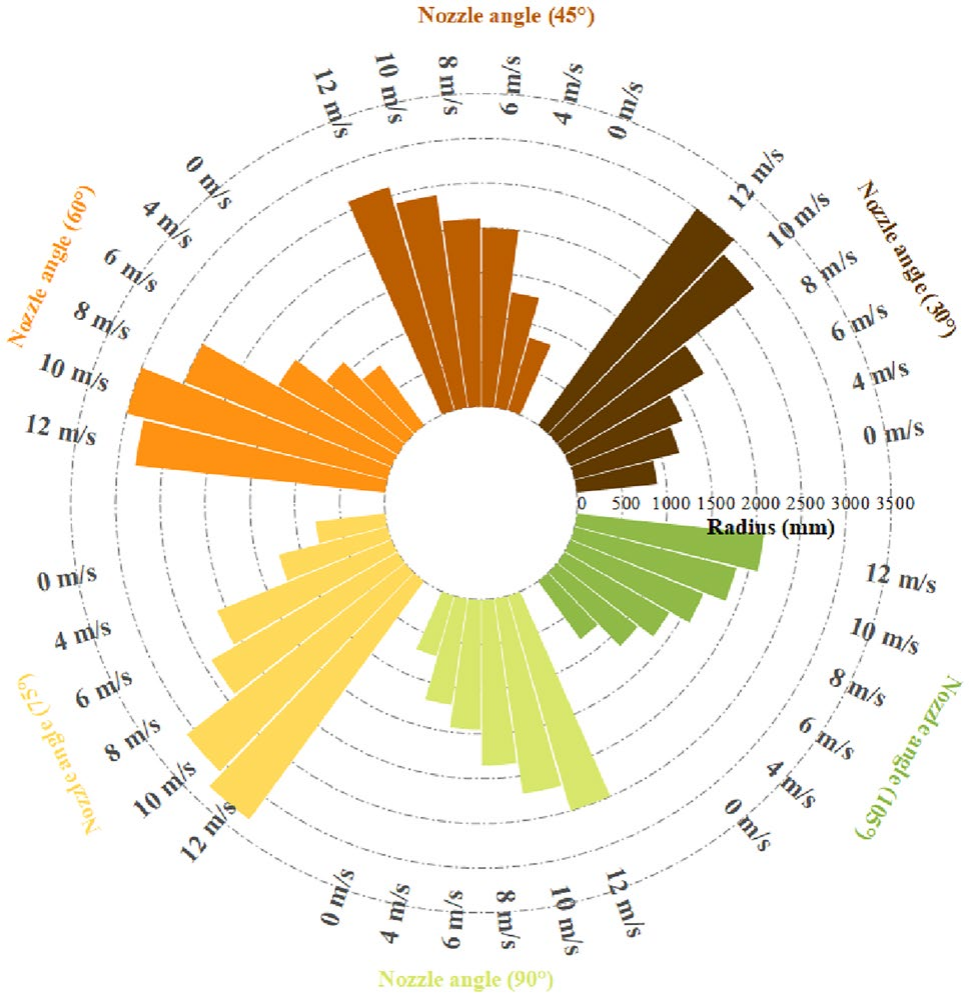
Variation law of fog screen coverage.

**Figure 17:**
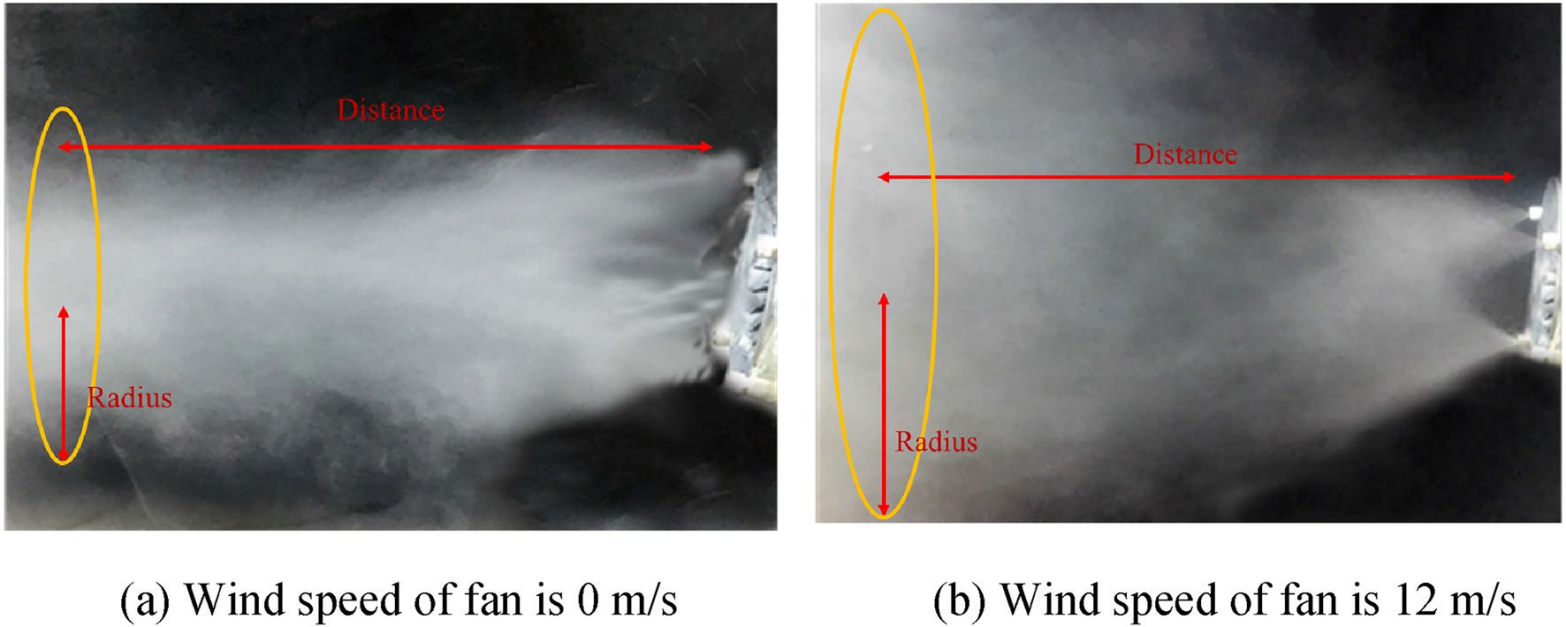
Spray effect picture at nozzle angle of 75° and wind speeds of 0 m/s and 12 m/s.

As indicated in Table 2, showcasing the spray coverage under various fan speeds and nozzle installation angles, Fig. 15 depicts the changes in the effective spray distance of the fog screen concerning wind speed and spray angle, while Fig. 16 illustrates the alterations in fog screen coverage with respect to wind speed and spray angle. After analysis, it was found:

① When the fan operates at a wind speed of 0 m/s, it is essentially equivalent to traditional high-pressure spray. In this scenario, when the spray angle increases to 90°, the effective spray distance expands, while the thickness and height of the fog curtain undergo minimal changes. These findings suggest that for traditional high-pressure spray, the maximum fog curtain coverage is achieved with a 90° nozzle angle.
② When the fan is utilized for vortex spray and the nozzle angle ranges from 30° to 75°, the experimental results align with the simulation results. As the fan's wind speed increases from 0 to 12 m/s, the fog curtain becomes thinner with increasing wind speed, while its width and height gradually increase. Figure 17 illustrates the spray effect with a 75° nozzle angle when the fan operates at wind speeds of 0 m/s and 12 m/s.
③ With the nozzle angle set at 75° and the fan's wind speed reaching 12 m/s, the fog screen coverage area measures 380 mm in width and 3300 mm in height. When the nozzle angle shifts to 90°, the fog screen coverage decreases compared to the 75° angle, and the smallest coverage is observed with a 105° nozzle angle. Therefore, when the nozzle installation angle is 75° and the fan operates at 12 m/s, the vortex spray achieves maximum fog screen coverage.
④ When the fan's wind speed reaches 12 m/s, the wind speed at the vortex spray head outlet, as measured by the anemometer, is 29.7 m/s, which is closely aligned with the experimental result of 30 m/s. This validation demonstrates the accuracy of the simulation data.

**Table 2:** Spray coverage scale.

Wind speed of fan (m/s)	30° spray coverage (distance × radius mm)	45° spray coverage (distance × radius mm)	60° spray coverage distance × radius mm)	75° spray coverage (distance × radius mm)	90° spray coverage (distance × radius mm)	105° spray coverage (distance × radius mm)
0	2400 * 900	2600 * 850	2800 * 830	3000 * 780	3200 * 700	2900 * 800
4	800 * 1200	850 * 1300	950 * 1120	1000 * 1250	950 * 1200	800 * 1150
6	700 * 1350	800 * 2000	800 * 1540	700 * 2100	750 * 1450	650 * 1350
8	600 * 1800	700 * 2100	700 * 2500	550 * 2400	500 * 1850	400 * 1600
10	500 * 2800	600 * 2400	600 * 3000	400 * 3100	300 * 2200	200 * 1860
12	450 * 3000	500 * 2600	450 * 2800	380 * 3300	300 * 2500	100 * 2100

### Experiment on variation of droplet size in a vortex aerodynamic fog screen

**Figure 18:**
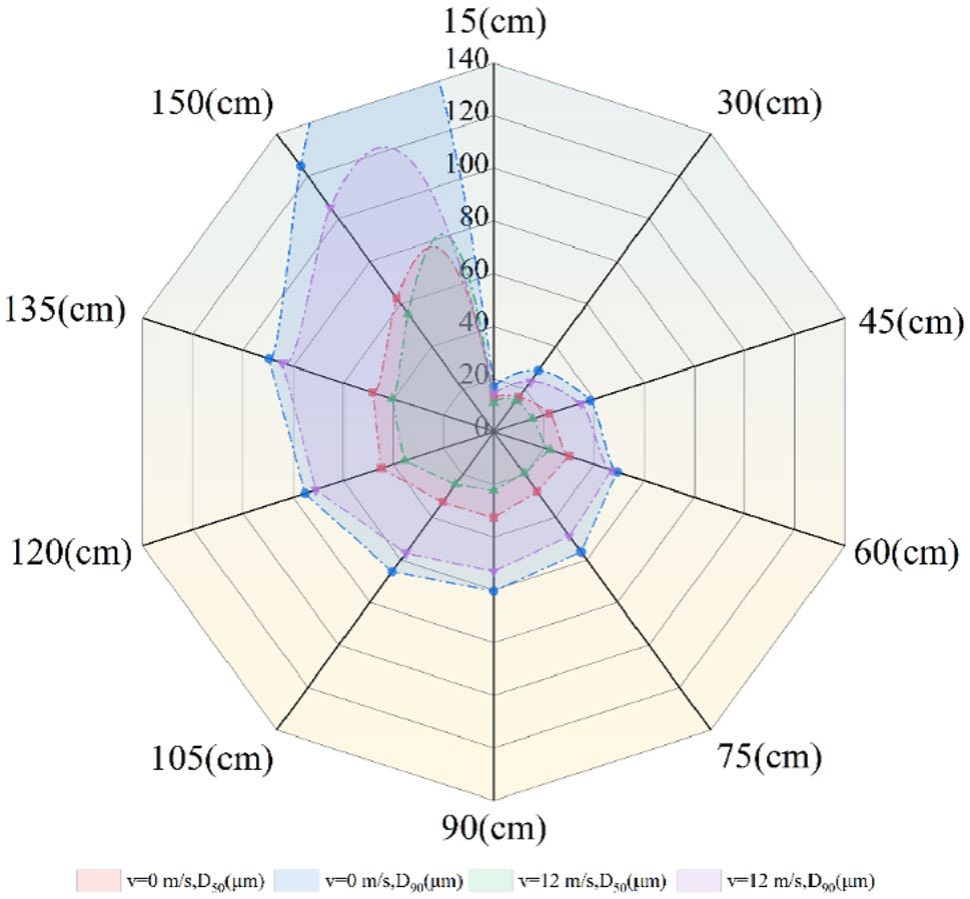
Comparison of particle size between vortex pneumatic fog screen and traditional high-pressure spray.

In spray particle size testing, d_50_ and d_90_ are crucial parameters that describe the distribution of particle sizes. The d_50_ value is an indicator of the central tendency of spray particle sizes, typically used to describe the median particle size. The d_90_ value is used to assess the distribution of larger particles within the spray, indicating the extent to which larger particles are present. We conducted spray experiments using the vortex pneumatic fog screen dust control device under two conditions: with the water supply pressure set to 8 MPa, the nozzle installation angle at 75°, and the fan operating at wind speeds of 12 m/s and 0 m/s. The variations in droplet diameter with distance are presented in Table 3. Based on the data in Table 3, we compared the droplet sizes produced by the vortex pneumatic fog screen, as depicted in Fig. 18, with those generated by traditional high-pressure spray.

**Table 3:** Spray coverage scale.

Spray distance/(cm)	Indicates droplet size value when fan supply wind speed is 12 m/s		Indicates droplet size value when fan supply wind speed is 0 m/s (traditional high-pressure spray)	
	D_50_/(μm)	D_90_/(μm)	D_50_/(μm)	D_90_/(μm)
15	10.895	14.564	13.105	17.253
30	14.416	23.636	16.235	28.684
45	15.485	34.640	22.262	38.351
60	22.456	47.022	30.216	49.348
75	19.517	48.908	27.834	56.286
90	22.283	52.796	32.564	60.437
105	24.610	56.800	32.948	65.447
120	35.642	70.852	44.842	75.277
135	40.415	84.263	48.275	89.646
150	55.426	105.246	62.474	124.638

**Figure 19:**
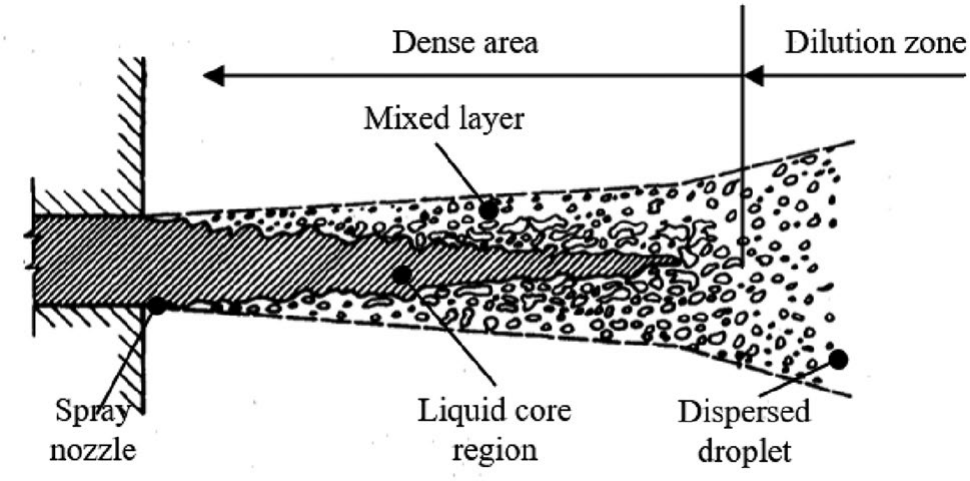
Division of spray jet region.

As depicted in Table 3 and Fig. 18, the droplet size of the vortex pneumatic fog screen and traditional high-pressure spray increases with increasing injection distance, but the droplet diameters D_50_ and D_90_ of the vortex pneumatic fog screen are always shorter than those of the traditional high-pressure spray. This is because the vortex air flow contributes to the secondary breakup of liquid droplets. A more detailed explanation is as follows: the atomization breaking process of the jet can be summarized as the disturbance breakup caused by the turbulent vortex, which is mostly the formation process of the liquid core. According to the division of the jet spray field, the atomized jet region from the nozzle can be divided into the dense zone and the dilution zone, and the spray jet region is divided as shown in Fig. 19. After the droplet is ejected, the droplet is broken by the impact and shear of the jet gas and is ejected in the form of a liquid wire or liquid line. Due to air disturbance, the droplet expands sharply until it breaks, and fine droplets are formed after a breakup. These droplet particles are impacted by the vortex airflow generated by the vortex fog curtain generator, the force and acceleration of the droplets in the dilution zone, secondary atomization such as collision or fragmentation and merging between the droplets, and further secondary breakup that occurs to form new and finer droplet particles.

Notably, within a spray distance of 60 cm, the vortex pneumatic fog screen is close to the traditional high-pressure spray droplet size D_90_. When the spray distance is 60–120 cm, there is a great difference in droplet size D_90_ between them. This is due to the gradual formation of vortex airflow after 60 cm and the gradual enhancement of the crushing effect on droplets. When the spray distance is greater than 120 cm, the difference between droplet sizes D_90_ and D_50_ decreases. Therefore, it can be seen that the best distance for secondary crushing of droplets by vortex air flow is 60–120 cm.

It's worth noting that the results show that the smaller the droplet diameter, the better the capture effect of respirable dust^45^. When the spray distance of the vortex pneumatic fog screen is greater than 105 cm, D_90_ is also greater than 100 μm. At the same time, considering the best distance of secondary crushing of liquid droplets by vortex air flow and the capture effect of respirable dust, approximately 90 cm is selected as the best dedusting distance of the vortex pneumatic fog screen.

### Experiment on effect of dust reduction

**Figure 20:**
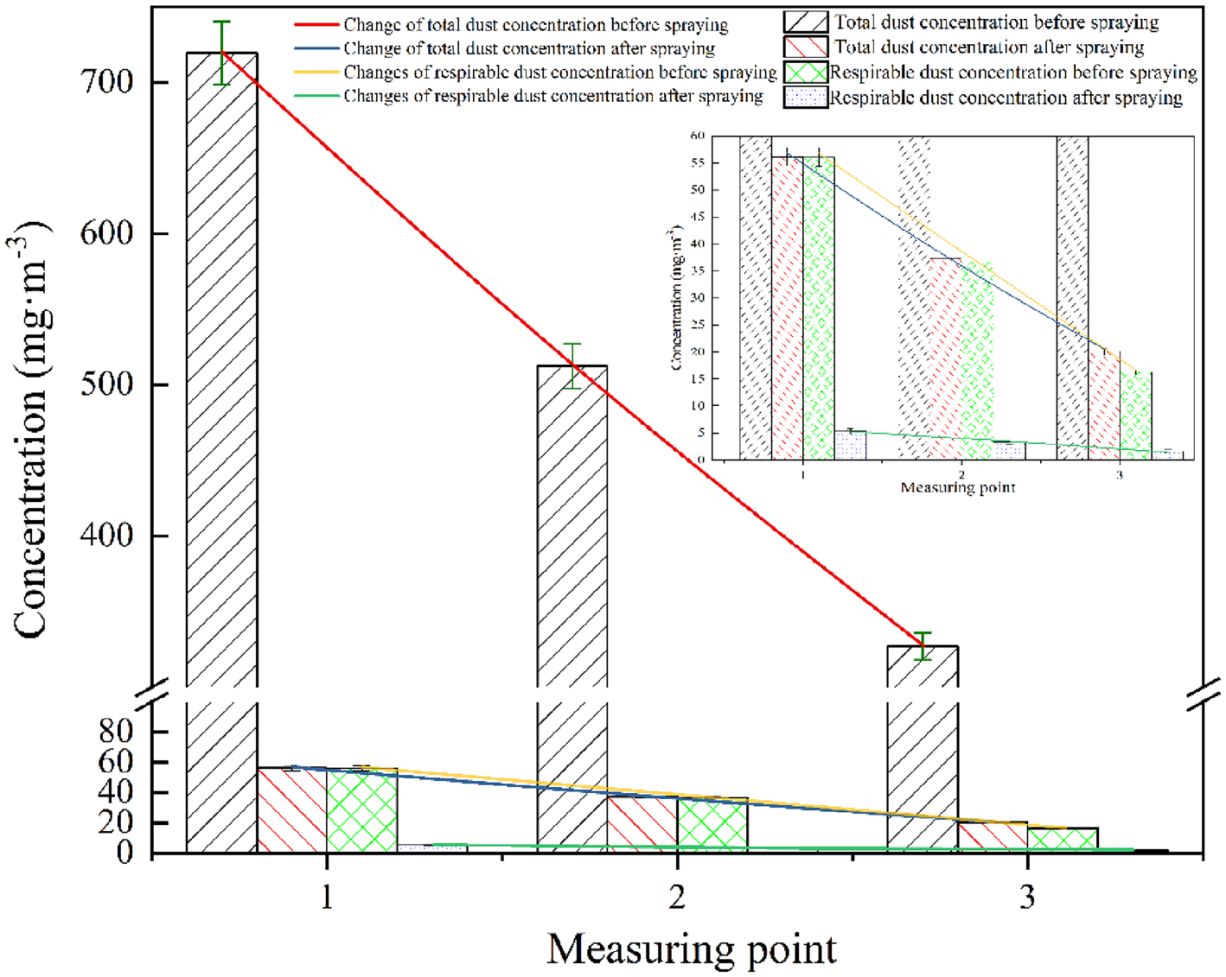
Dust concentration change.

Analyzing the dust concentration test data in Table 4, we observe changes in dust concentration before and after activating the dust removal device, as illustrated in Fig. 20. Our analysis of the data reveals the following:

**Table 4:** Test data of dust concentration.

Measuring point	Dedusting device is not turned on (mg m^−3^)		Dedusting device turned on (mg m^−3^)		Dust reduction efficiency (%)	
	Total dust	Respirable dust	Total dust	Respirable dust	Total dust	Respirable dust
1	719.32	56.21	56.10	5.34	92.20	90.50
2	512.27	37.35	36.76	3.32	92.82	91.11
3	326.88	20.11	16.31	1.69	95.01	91.60

Upon activation of the vortex pneumatic fog screen dust control device, its dust capture capability is significantly enhanced, particularly at the measurement point closest to dust source 1. The total dust concentration drops from 719.32 to 56.10 mg/m^3^, achieving a dust reduction efficiency of 92.20%. Furthermore, the concentration of respirable dust falls from 56.21 to 5.34 mg/m^3^, achieving a dust reduction efficiency of 90.5%. These figures provide compelling evidence that this device effectively prevents the dispersion of fine dust particles throughout the entire roadway.

It is worth noting that the dust reduction efficiency of traditional high-pressure spray systems typically ranges from 60 to 80%. However, upon activating the vortex spray dust control device, the dust reduction efficiency increases by 5% compared to traditional high-pressure spray. Evidently, the vortex spray dust control device exhibits a superior dust-capturing capacity. This enhanced performance can be attributed to the rotating nature of the spray within the vortex flow, leading to a spiral trajectory and an increased droplet-to-dust collision probability. Simultaneously, the high-speed movement of the vortex flow generates a negative pressure zone at its center, affecting the dust particles that escape initial capture along the fog curtain's periphery.

Additionally, activating the vortex pneumatic fog screen dust control device, there is a noticeable reduction in dust concentration. The average dust reduction efficiency for respirable dust stands at 91.07%, while the overall dust reduction efficiency reaches 93.34%. With the device in operation, it establishes a protective fog curtain, effectively separating the dust-generating surface from personnel on both sides of the fog curtain.

## Conclusion

Based on a mature dust removal mechanism of wind curtains and high-pressure spray, combined with information from dust control studies by foreign and domestic scholars on coal mining faces, a set of vortex pneumatic fog screen dust control devices suitable for fully mechanized mining faces was developed. A simulation roadway was set up to test the dust removal performance of the device and compare it with CFD numerical simulations. The following conclusions can be summarized.

The utilization of vortex pneumatic fog screen dust control technology results in significantly smaller droplet sizes, notably when compared to traditional high-pressure spray methods. The device achieves its peak performance when the water supply pressure of the pump is set at 8 MPa, the fan operates at a wind speed of 12 m/s, and the nozzle is installed at a 75° angle. Under these conditions, the device generates a spiral fog curtain with a spray coverage of 380 mm in width and 3300 mm in height, effectively suppressing dust emissions originating from one side of the air curtain.

To optimize the device's dust removal capabilities, an approximate dedusting distance of 90 cm was chosen, taking into consideration the secondary crushing of liquid droplets by the vortex airflow and the device's efficacy in capturing respirable dust. This configuration yields a D_50_ droplet diameter of 32.564 µm and a D_90_ droplet diameter of 60.437 µm.

Based on the results of dust reduction experiments, the application of the vortex pneumatic fog screen dust control device results in an average dust reduction efficiency of 91.07% for respirable dust and 93.34% for all dust types. This technology enhances the distance over which droplets effectively collide with dust and captures escaped respirable dust through negative pressure entrainment.

Overall, the development of the vortex pneumatic fog screen dust control technology was achieved through a combination of theoretical analysis and experimental verification. Although the model device was continuously refined during the experiment, it has not yet been applied in the operational context of an underground fully mechanized heading face. Future research endeavors will focus on enhancing the device and conducting in-depth investigations into the impact of nozzle aperture diversity and the collision and condensation processes of simulated particles within the vortex fog screen dust control technology.

## Data Availability

The data that support the findings of this study are available from the corresponding author, Ping Chang, upon reasonable request.
